# Hematopoietic stem and progenitor cells as a reservoir for trained immunity

**DOI:** 10.7554/eLife.106610

**Published:** 2025-09-04

**Authors:** Brandon T Tran, Vidthiya Jeyanathan, Ruoqiong Cao, Eva Kaufmann, Katherine Y King

**Affiliations:** 1 Department of Pediatrics, Division of Infectious Diseases, and Stem Cells and Regenerative Medicine Center, Baylor College of Medicine and Texas Children’s Hospital Houston United States; 2 Program in Cancer and Cell Biology, Graduate School of Biomedical Sciences, Baylor College of Medicine Houston United States; 3 Department of Biomedical and Molecular Sciences, Queen’s University Kingston Canada; 4 Program in Immunology and Microbiology, Graduate School of Biomedical Sciences, Baylor College of Medicine Houston United States; Murdoch Childrens Research Institute Melbourne Australia; The University of Tokyo Tokyo Japan

**Keywords:** innate immune memory, trained immunity, hematopoietic stem cells, inflammation, epigenetics, metabolism, hematopoietic progenitor cells

## Abstract

Human and murine studies reveal that innate immune cells are able to mount enhanced responses to pathogens after primary inflammatory exposure. Innate immune memory has been shown to last for months to years, longer than the lifespan of most innate immune cells. Indeed, long-lived hematopoietic stem and progenitor cells (HSPCs) serve as a cellular reservoir for innate immune memory. In this review, we summarize the evidence that innate immune memory is epigenetically encoded in HSPCs, and we consider whether HSPC subpopulations with differentiation bias, cell autonomous epigenetic reprogramming, or both features underlie the phenomenon of central trained immunity. We further profile the significant implications of central trained immunity in stem cell transplant, aging, inflammatory diseases, and vaccination strategies for the future.

## Innate immune memory: a new paradigm in immunology

‘Trained immunity’, or ‘innate immune memory’, describes the concept that innate immune cells, such as macrophages (Kaufmann et al., 2018; Yao et al., 2018), neutrophils (Kalafati et al., 2020), and NK cells (Verma et al., 2017)**,** gain enhanced function after a primary encounter with an immunological stimulus (Netea et al., 2020). While adaptive immune memory is achieved by recombination of antigen receptors followed by positive and negative selection, trained immunity is generated through epigenetic modifications and metabolic reprogramming incurred upon inflammatory stimulation (Quintin et al., 2012; Cirovic et al., 2020). Trained immunity can be induced through a variety of infections, vaccines, and adjuvants such as Bacillus Calmette–Guérin (BCG) and the fungal cell wall component beta glucan (β-glucan), or inflammatory stimuli released during autoimmune diseases. Trained immunity initiated by these stimuli can enhance cross-protective host immunity against infections or cancer (Kalafati et al., 2020; Hersh et al., 1977; van Puffelen et al., 2020; Larsen et al., 2020b; Mills et al., 2024; Walk et al., 2020). Innate immune memory has also been associated with maladaptive effects such as periodontitis, arthritis, and long COVID (Cheong et al., 2023; Christ et al., 2018, Li et al., 2022). Indeed, some attempts to use trained immunity to protect against infection have resulted in counterproductive hyperinflammation (Walk et al., 2019). Thus, while trained immunity can serve as a powerful tool to improve host immunity, it also has the capacity to confer a detrimental burden on patient health.

In humans, trained immunity after BCG vaccination was found to last for over 1 year in circulating monocytes (Kleinnijenhuis et al., 2014). These long-term effects were surprising since circulating monocytes have a half-life of only a few days in humans and mice (Patel et al., 2017; Teh et al., 2019). As circulating innate immune cells lack self-renewal capacity and are primarily short-lived, investigators have turned to hematopoietic stem and progenitor cells (HSPCs), the long-lived and self-renewing progenitors of innate immune cells (see Box 1), as the potential reservoir for trained immunity (Orkin and Zon, 2008). Possessing both multilineage differentiation potential and self-renewal capacity, HSPCs are known to respond to environmental cues such as inflammation triggered by infection, diet, or aging (King and Goodell, 2011; Takizawa et al., 2012; Pietras, 2017; Ding et al., 2021). Indeed, HSPCs express Toll-like receptors and other pathogen-associated molecular pattern recognition and cytokine receptors that equip them to detect and respond to inflammatory stimuli. Upon detection of external stressors, HSPCs undergo emergency myelopoiesis, thereby directly contributing to the immune response (Cao et al., 2024).

Box 1. **Hematopoietic stem and progenitor cell (HSPC) subsets.**

**Figure 1.**
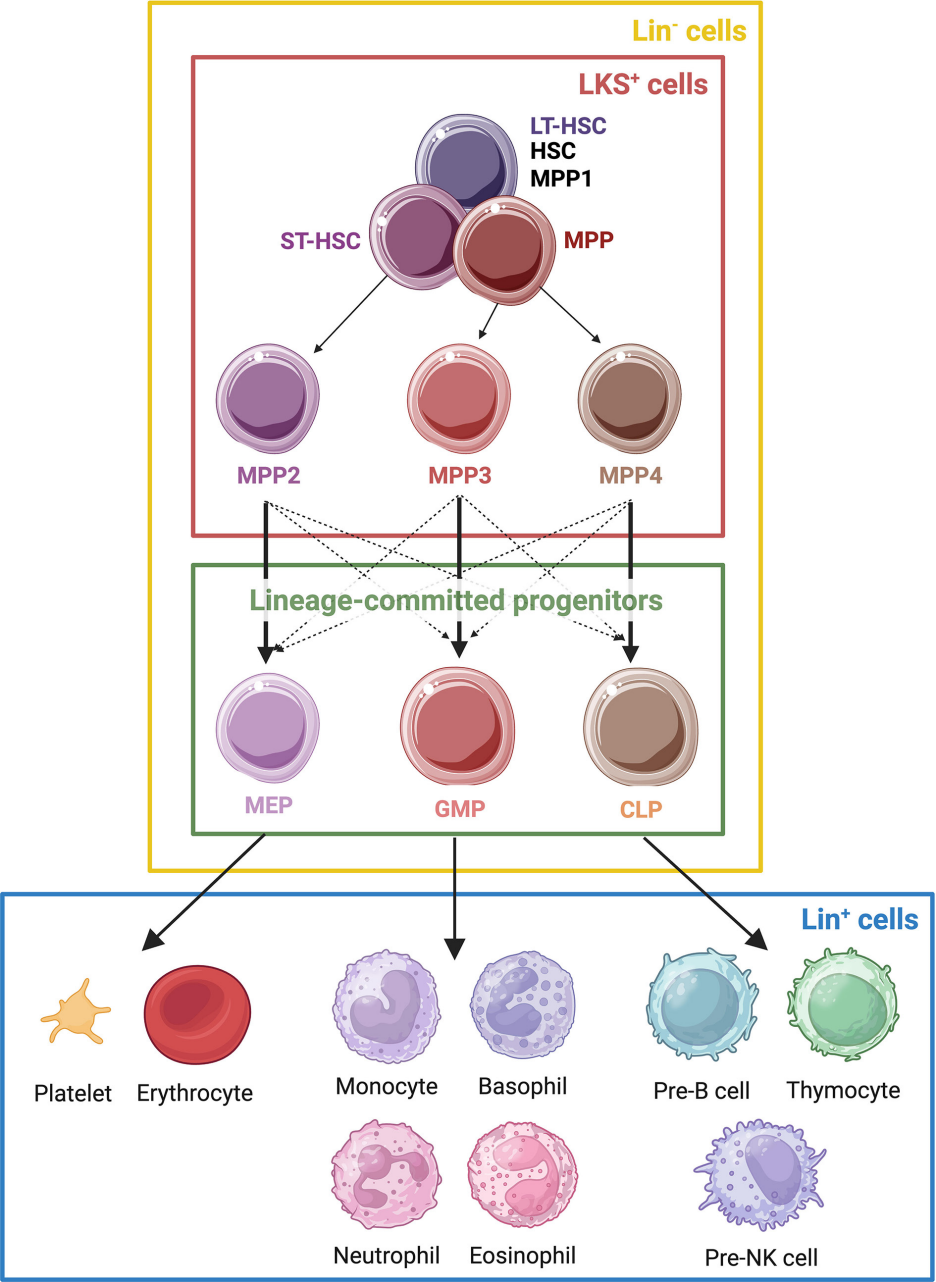
Hierarchical model of hematopoiesis. Hematopoiesis is often depicted as a linear cell fate tree; however, recent investigation has shown developmental relationships between the various hematopoietic lineages. The LKS cell population comprises long-term hematopoietic stem cells (LT-HSCs), short-term HSCs (ST-HSCs), and multipotent progenitors (MPPs) in various stages. HSCs give rise to MPPs, with MPP1 representing early multipotent progenitors that are a metabolically active form of the most quiescent HSCs with the capacity for multi-lineage potential. Further downstream are specific multipotent progenitors (MPPs) which give rise to lineage-committed progenitors that are restricted in their differentiation potentials. MPP2 is biased to give rise to megakaryocyte–erythrocyte progenitors (MEPs), MPP3 to granulocyte–monocyte progenitors (GMPs), and MPP4 to common lymphoid progenitors (CLPs). While each of these multipotent progenitors is biased toward the production of specific lineage-committed progenitors, they can be skewed to favor the expansion of others, depending on the nature of the bone marrow microenvironment and timing of an insult. Lineage-committed progenitors develop into mature blood cells: MEP into platelets and erythrocytes, GMP into monocytes, basophils, neutrophils, and eosinophils, and CLP into pre-NK cells, pre-B cells, and thymocytes.

HSPCs are composed of a heterogeneous pool of stem and progenitor cells, each with varying capacity for multipotency and self-renewal. HSPCs include long-term HSCs (LT-HSCs), short-term HSCs (ST-HSCs), multipotent progenitors (MPPs), common myeloid progenitors (CMPs), megakaryocyte–erythrocyte progenitors (MEP), granulocyte–monocyte progenitors (GMPs), and common lymphoid progenitors (CLPs) (Figure 1). While cell types are ultimately defined by their functional properties, they are often phenotypically characterized by cell surface marker expression.

LSK cells and lineage-committed progenitors compose the lineage-negative population, indicating the absence of lineage-specific markers on fully differentiated cells, such as myeloid and lymphoid cells. LT-HSCs are the most primitive HSCs, retaining the capability to self-renew over an individual’s entire lifespan. LT-HSCs were first characterized through studies that demonstrated their ability to regenerate the bone marrow of irradiated mice over an extended period of time (Nakauchi et al., 1999). The widely used surface markers associated with this cell population are Lin^−^Sca1^+^c-Kit^+^CD34^−^CD150^+^CD48^−^Flt3^−^ (Nakauchi et al., 1999; Yang et al., 2005; Challen et al., 2009; Rathinam et al., 2011; Wang et al., 2013; Belle et al., 2016). ST-HSCs derive from LT-HSCs but lack the high self-renewal capacity of their upstream precursors. Despite this, they retain their multipotency and act as the primary source of key effector cells that execute the rapid and robust response central to trained immunity and contribute to hematopoiesis over the span of weeks to months (Challen et al., 2009). ST-HSCs share many surface markers with LT-HSCs, albeit with subtle differences. In mice, ST-HSCs are typically Lin^−^Sca1^+^c-Kit^+^CD34^+^CD150^+^CD48^+^Flt3^−^ (Nakauchi et al., 1999; Yang et al., 2005; Challen et al., 2009; Rathinam et al., 2011; Wang et al., 2013; Belle et al., 2016).

MPPs are downstream of LT-HSCs and ST-HSCs but are still considered LSK cells. They give rise to all major blood cell lineages, including erythroid, myeloid, and lymphoid cells. Phenotypically, they are similar to ST-HSCs (Yang et al., 2005; Wang et al., 2013). These cells are critical bridges between HSCs and lineage-committed progenitors and can be further differentiated into distinct subpopulations. MPP1s are highly similar to LT-HSCs, but metabolically more active and have self-renewal capacity. They are primarily biased toward lymphoid and myeloid lineages and are typically marked by CD150^+^CD48^−^CD34^+^Flt3^−^ surface expression (Pietras et al., 2015; Rathinam et al., 2011; Wang et al., 2013; Belle et al., 2016; Wilson et al., 2008; Cabezas-Wallscheid et al., 2014). MPP2s are more restricted than MPP1s and are biased toward differentiation into megakaryocytes and erythrocytes. This subset is marked by CD150^+^CD48^+^CD34^+^Flt3^−^ surface expression (Rathinam et al., 2011; Wang et al., 2013; Belle et al., 2016; Wilson et al., 2008; Cabezas-Wallscheid et al., 2014). MPP3s give rise to myeloid cells, with a bias toward granulocytes and monocytes. These cells are characterized by CD150^−^CD48^+^CD34^+^Flt3^−^ surface expression (Rathinam et al., 2011; Wang et al., 2013; Belle et al., 2016; Wilson et al., 2008; Cabezas-Wallscheid et al., 2014). MPP4s are primarily lymphoid-biased and are typically marked by CD150^−^CD48^+^CD34^+^Flt3^+^ surface expression (Rathinam et al., 2011; Wang et al., 2013; Belle et al., 2016; Wilson et al., 2008; Cabezas-Wallscheid et al., 2014).

The identification and isolation of HSCs are in most part dependent on their surface marker expression, but the inconsistent use of surface markers in categorizing different HSC subsets sparks debate. For instance, some authors label ST-HSCs as positive for CD150 and CD48 (Rathinam et al., 2011; Wang et al., 2013), whereas others label them as negative for those surface markers (Pietras et al., 2015; Sawai et al., 2016). Differences in experimental models, such as mouse strains, method of isolation (flow cytometry versus magnetic bead separation), age of the cells, or physiological state of the organism complicate the unification of defining HSCs, potentially through up- and downregulation of surface marker expression (Morrison and Scadden, 2014). Nevertheless, the consensus paper published in 2021 by Challen et al. brings together most of these markers and categories (Challen et al., 2021). Further marker description will enhance the potential to discriminate different subpopulations. In light of discrepancies in the field, naming of all markers for definition of certain populations remains of significant importance for ongoing use and extrapolation of experimental results. Coming to a consensus as well as incorporating functional assays for identification of these cell populations will ultimately be crucial for ongoing HSC research and its clinical applications.

Markers of HSPCs differ substantially between mice and humans. Human HSCs are defined by the cell surface markers CD34^+^CD38^−^CD90^+^CD45RA^−^ (Cimato et al., 2016). Unlike their mouse counterparts, the nomenclature of human HSCs is less debated due to consistent identification of surface markers, which remain mostly stable, and functional assays utilized in human studies (Anjos-Afonso and Bonnet, 2023). Human LT-HSCs are classified as Lin^−^CD34^+^CD38^−^CD90^+^CD45RA^−^CD49f^+^, which give rise to ST-HSCs that express Lin^−^CD34^+^CD38^−^CD90^−^CD45RA^−^CD49f^−^ (Rix et al., 2022). MPP-F1 (CD34^+^CD38^−^CD90^−^CD45RA^−^CD71^−^CD110^−^) and MPP-F2/3 (CD34^+^CD38^−^CD90^−^CD45RA^−^CD71^+^) are three cellular fractions that derive from ST-HSCs, of which the former retain greater multilineage differentiation potential (Notta et al., 2016).

The potential for HSPCs to serve as a memory reservoir for trained immunity has been described in both murine (Kaufmann et al., 2018; Mills et al., 2024; Mitroulis et al., 2018; Khan et al., 2020; Kain et al., 2023; Zhu et al., 2024; Kalafati et al., 2020; de Laval et al., 2020; Table 1) and human studies (Cirovic et al., 2020; Cheong et al., 2023; Sun et al., 2024). Experiments in which memory phenotypes were transferred via HSPCs from trained donors into irradiated recipients have demonstrated the sufficiency of reprogrammed HSPCs to generate innate immune memory in circulating immune cells (Kaufmann et al., 2018; Mitroulis et al., 2018; Khan et al., 2020; Kain et al., 2023; de Laval et al., 2020). Long-term reprogramming has been observed in mouse studies of hemozoin-dependent *Plasmodium* infection (Zhu et al., 2024) and in longitudinal studies in humans trained with BCG vaccination (Cirovic et al., 2020; Sun et al., 2024) or long COVID (Cheong et al., 2023). Notably, innate immune memory has also been observed in non-immune cells, such as epidermal stem cells (Naik and Fuchs, 2022; Larsen et al., 2020a) and embryonic fibroblasts (Kamada et al., 2018). Like immune cells, these structural cells exhibit heightened transcriptional responses following an initial training period of inflammatory exposure and recovery. A common theme is that cells harboring ‘memory’ must either be self-renewing, long-lived, or both. At the very least, these cells must be able to survive the initial insult for the reprogramming to persist.

**Table 1.** Hematopoietic stem and progenitor cell (HSPC) populations mediating trained immunity phenotypes.

Cell type	TI experimental endpoint	Inducing stimulus	TI phenotype	Citations
Whole bone marrow	20 weeks	BCG	Protection from *M. tb* infection	Kaufmann et al., 2018
	1 year	*M. tb*	Susceptibility to *M. tb*	Khan et al., 2020
cKit enriched	16 weeks to >1 year	*M. avium*	Cross protection	Kain et al., 2023
		H1N1		
LSK	13 weeks and secondary transplant	LPS	Protection from *P. aeruginosa*	de Laval et al., 2020
LT-HSC	13 weeks and secondary transplant	LPS	Protection from *P. aeruginosa*	de Laval et al., 2020
	8 months	*Fasciola hepatica* excretory–secretory products (FHES)	Reduced susceptibility to induction of EAE	Cunningham et al., 2021
	1 year	*M. tb*	Inhibit trained immunity	Khan et al., 2020
ST-HSC/MPP3	4 weeks	BCG	↑ ST-HSC, MPP3	Kaufmann et al., 2018
		*M. tb*	↓ ST-HSC, MPP3	Khan et al., 2020
		Heme	↑ ST-HSC, MPP3	Jentho et al., 2021
		Western diet	↓ MPPs	Christ et al., 2018
GMPs	1 week	β-Glucan	Anti-tumor immunity	Kalafati et al., 2020
	1 year	COVID	Long-term inflammation	Cheong et al., 2023
	12 weeks	Sepsis	Post-sepsis immunosuppression	Bomans et al., 2018

The mechanism by which HSPCs encode memory is the subject of intense investigation. Recent studies indicate two key findings: (1) inflammatory responses by HSPCs in the bone marrow are highly heterogeneous (Kain et al., 2023; Arts et al., 2018) and (2) epigenetic changes in response to infection affect HSPC quiescence and differentiation (Sun et al., 2024; de Laval et al., 2020; Hormaechea-Agulla et al., 2021). These observations raise the potential that inflammatory stimuli can affect innate immune function via selective activation, involving proliferation and differentiation, and epigenetic reprogramming of HSPC subsets, thereby influencing lineage fate determination and reshaping immune cell populations (Johansson et al., 2023).

Overall, the concept that HSPCs in the bone marrow, where 90% of HSPCs are found, are a long-term reservoir for innate immune memory is termed ‘central trained immunity’ to distinguish it from reprogramming in peripheral cell populations (Netea et al., 2020; Mende and Laurenti, 2021). In this review, we focus on the emerging evidence and mechanisms by which HSPCs encode long-term innate immune memory.

## Investigating the role of hematopoietic stem cells in central trained immunity

Hematopoietic stem cells (HSCs) are the adult stem cells responsible for lifelong production of blood, including immune cells. HSCs are positioned at the apex of the hematopoietic hierarchy and possess the highest multipotency and self-renewal potential (Figure 1, see Box 1). Transcriptional studies have demonstrated that inflammation promotes differentiation and loss of self-renewal potential of HSCs (Pietras, 2017; Matatall et al., 2014; Baldridge et al., 2010), leading to their eventual exhaustion (Matatall et al., 2016). Like monocytes and macrophages, HSCs express receptors for inflammatory cytokines and pathogen-associated molecular patterns such as LPS (Baldridge et al., 2010; Nagai et al., 2006; Essers et al., 2009). They can directly respond to these stimuli by producing more inflammatory cytokines, activating downstream inflammatory response pathways such as JAK–STAT signaling, and shifting metabolic pathways through mTOR signaling (Cheng et al., 2014; Mills et al., 2024; Kain et al., 2023; Baldridge et al., 2010). Notably, innate immune function responses like antigen presentation, specifically of MHC class II genes like *H2-Ab1*, can be observed in HSCs in several contexts including *M. avium* infection (Hormaechea-Agulla et al., 2021), LPS stimulation (Hernández-Malmierca et al., 2022), and autoimmune disease models mimicking systemic lupus erythematosus (Mills et al., 2024). Thus, despite their disparate position in the hematopoietic hierarchy, HSCs and innate immune cells share many common biological processes. This observation raises the possibility that inflammatory stimuli activate and reprogram transcription networks that are directly passed down to trained immune cell progeny.

**Figure 2.**
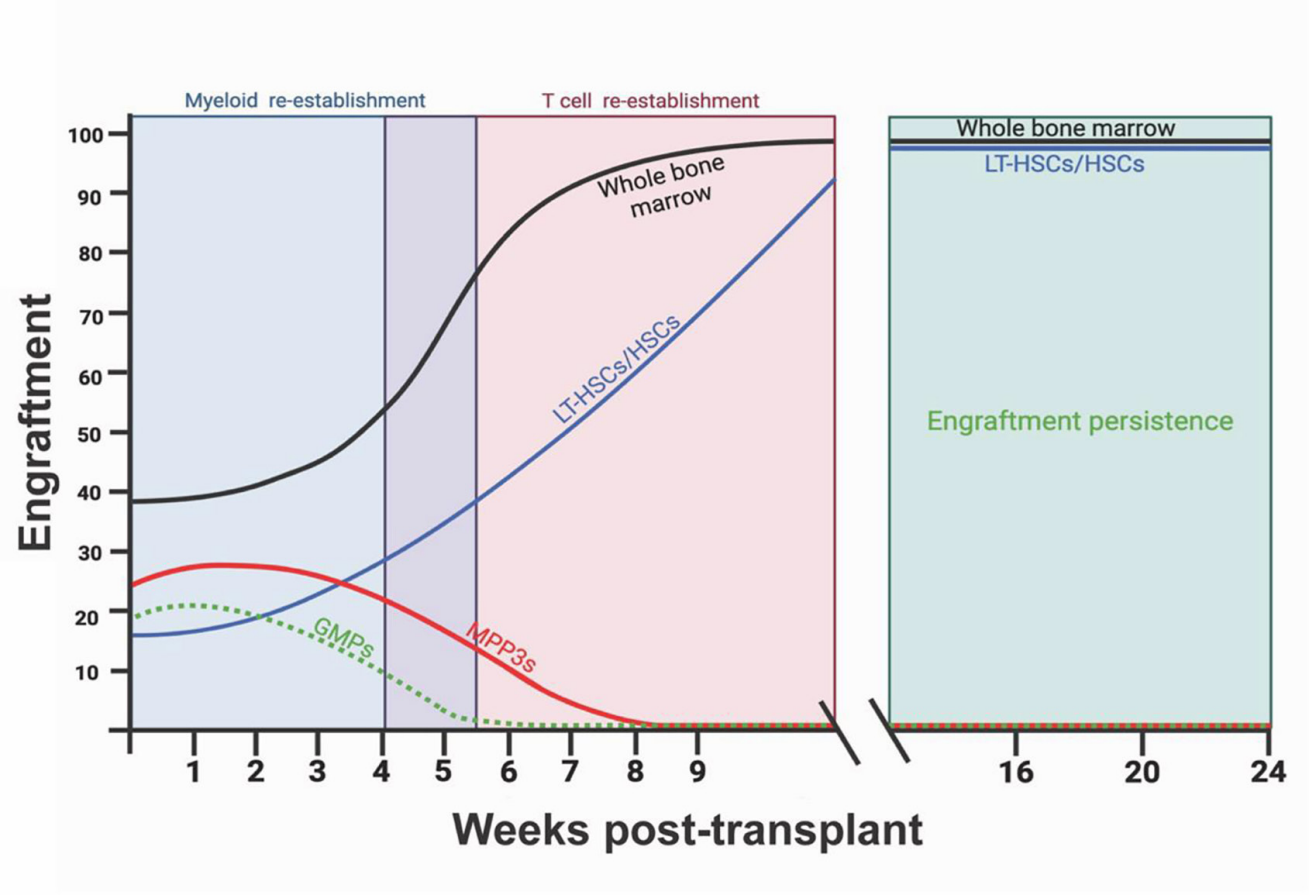
The engraftment potential of hematopoietic stem and progenitor cell (HSPC) subpopulations in transplant mouse models. Murine transplant studies have been used to determine engraftment capacity of hematopoietic cells. Whole bone marrow (black line) can be transplanted into irradiated recipients to fully reestablish the hematopoietic system. Myeloid cells such as neutrophils and macrophages are the first to develop in the first few weeks post-transplantation, followed by lymphoid cells (Pietras et al., 2015; Ogonek et al., 2016). Hematopoietic stem cells (HSCs) are considered the main contributors to the long-term engraftment observed in hematopoietic transplant models. A single HSC (blue) can reconstitute the mouse hematopoietic system and can be serially transplanted for continued long-term hematopoiesis (Pietras et al., 2015; Oguro et al., 2013; Spangrude et al., 1988). Unlike HSCs and whole bone marrow, myeloid-biased downstream progenitors, such as multipotent progenitors (MPPs) (red), have been reported to sustain multilineage hematopoiesis for 4 weeks before their exhaustion and disappearance (Pietras et al., 2015; Oguro et al., 2013). The persistence of granulocyte–monocyte progenitors (GMPs) alone is even shorter compared to MPP3s (labeled in dashed green) (Pietras et al., 2015; Säwen et al., 2018). Of note, barcoding studies have shown much longer multilineage hematopoiesis from MPPs and GMPs in native, non-transplant conditions (Shaban et al., 2025).

Methods to study HSC function have been developed over several decades. HSC transplantation studies in mice have been used to functionally define HSCs and study their role in various contexts, including trained immunity (Kaufmann et al., 2018; Mitroulis et al., 2018; Pietras et al., 2015; Ochando et al., 2023). While many different transplant strategies exist (Kang et al., 2024; Yeung et al., 2024; Chanut et al., 2021), the most common ones use whole body irradiation to ablate fast cycling cells, clear the hematopoietic niche, and allow for successful engraftment of donor HSPCs. Donor HSPCs injected intravenously home to the bone marrow and produce blood for a period of time that is proportionate to their self-renewal potential (Pietras et al., 2015; Al-Amoodi et al., 2022). Multipotent progenitors and committed myeloid progenitors can produce blood, particularly myeloid cells, for up to several weeks, whereas long-term engraftment beyond 3 months is maintained by long-term HSCs (LT-HSCs) (Säwen et al., 2018; Oguro et al., 2013; Spangrude et al., 1988; Figure 2). This observation has led some to conclude that LT-HSCs must be the reservoir for long-term central trained immunity; however, our own work transplanting *M. avium*-trained LT-HSCs suggests LT-HSCs alone cannot confer central trained immunity-mediated host protection (Kain et al., 2023). In addition, studies of native hematopoiesis in the absence of transplantation have indicated a significant long-term contribution of multipotent progenitors to hematopoiesis (Sun et al., 2014; Busch and Rodewald, 2016; Pei et al., 2017; Cheng et al., 2020). This raises the possibility that multipotent progenitors may play a key role in central trained immunity. New *in vitro* culture methods like polyvinyl alcohol culturing of HSCs and/or downstream progenitors will enable researchers to assess the durability and relevance of epigenetic reprogramming in specific HSPC subpopulations (Wilkinson et al., 2019).

### Investigating central trained immunity in transplantation settings

In the first study to investigate the role of HSPCs in central trained immunity, we showed that reprogrammed HSPCs provide improved, non-specific host immunity to recipient mice. Specifically, transplant of whole bone marrow from BCG iv-vaccinated mice provided immunity against subsequent *Mycobacterium tuberculosis* (*M. tb*) challenge 14 weeks post-transplant (Kaufmann et al., 2018). Further refinement of transplant studies, such as transplanting lineage-negative, cKit+, Sca1+ cells (de Laval et al., 2020) or cKit-enriched cells (Kain et al., 2023) primarily composed of HSPCs, provided further evidence that primary transplant of trained HSPCs can improve host immunity in subsequent challenges. In studies using the related bacterium *M. avium* as the training agent, we found that similar host protection could persist for 1 year post-primary transplant (Kain et al., 2023). Importantly, studies have utilized both mycobacteria and LPS as training agents, suggesting diverse molecular avenues for training (de Laval et al., 2020; Kaufmann et al., 2022). Trained immunity-mediated host protection was also replicated in the setting of *Rag1*-deficient mice, indicating independence from the adaptive immune system (Quintin et al., 2012). We showed that *M. avium* training could confer cross-protective immunity against an antigenically distinct H1N1 influenza challenge, reinforcing the concept that trained immunity protection is not attributable to an adaptive immune response (Kain et al., 2023). Multiple studies support this finding (Kaufmann et al., 2022; Ciarlo et al., 2020; Khan et al., 2025).

### Secondary transplantation to enhance stringency of HSC memory studies

Serial transplantation is the most stringent functional test of HSC memory capacity, as it requires stem cells to self-renew and generate trilineage hematopoiesis across two or more rounds of transplantation. Serial transplantation of BCG-trained whole bone marrow over a 42-week time period conferred innate immune memory, while a maladaptive memory phenotype induced by *M. tb* likewise was transferred in this transplant model (Khan et al., 2020). Secondary transplantation of LPS-stimulated LSK cells 12 weeks apart showed that trained HSCs mediated decreased bacterial burden and increased survival in secondary recipients that had been challenged with lethal doses of *P. aeruginosa* (de Laval et al., 2020). These secondary transplant experiments, because they require two rounds of flow cytometric purification of HSPCs, significantly reduce the chance that trained immunity phenotypes are due to transmission of the original pathogen to recipient animals or due to transfer of long-lived adaptive immune cells. Altogether, these studies provide strong evidence that reprogramming of HSPCs results in robust and durable innate immune memory in murine models. However, more direct evidence of HSC involvement in trained immunity is needed, as very few studies to date have utilized direct transplant of purified HSCs, and LSK populations used for multiple studies include numerous progenitors other than HSCs (de Laval et al., 2020).

## The role of short-term HSCs (ST-HSCs) and multipotent progenitors (MPPs) in innate immune memory conservation

Transplant models typically involve pre-conditioning regimens such as irradiation or chemotherapy to enable engraftment of transplanted cells (Hasan et al., 2024). These harsh regimens induce systemic inflammation, immune cell activation, and disruption of the bone marrow niche, thereby introducing confounding variables to studies (Mitroulis et al., 2018). Transplant models also reduce the capacity to study contributions by MPPs or other progenitors which may be long-lived in native hematopoiesis but not after transplant, as mentioned above. While transplant models remain invaluable for addressing questions of cellular origin and lineage tracing, non-transplant approaches provide complementary insights by focusing on the intrinsic properties of HSPCs within a physiologically intact niche, offering a reproducible framework for studying trained immunity (Netea et al., 2020; Bloom et al., 2023).

Compared to LT-HSCs, ST-HSCs, and MPPs are thought to have a more limited lifespan of less than 6 months and typically contribute more transiently to hematopoiesis, rapidly cycling to replenish downstream progenitor populations (Sun et al., 2014; Busch and Rodewald, 2016; Pei et al., 2017; Cheng et al., 2020; Ema et al., 2014). ST-HSCs and MPPs retain multipotency, a high proliferation potential compared to LT-HSCs, and a strong commitment to lineage differentiation, serving as critical intermediates that amplify cell numbers and ensure robust generation of lineage-restricted progenitors, thereby supporting the rapid production of fully differentiated immune cells (Höfer and Rodewald, 2018). MPP2, MPP3, and MPP4 exhibit distinct lineage biases, with MPP2 favoring megakaryocytic/erythroid differentiation, MPP3 displaying a myeloid bias, and MPP4 predominantly contributing to lymphoid lineages (see Box 1; Pietras et al., 2015; Kang et al., 2023). Unlike LT-HSCs, ST-HSCs, and MPPs do not sustain hematopoiesis over extended periods of time following transplantation, making it difficult to study the contribution of these cells in transplant models.

Several non-transplant studies reveal a possible role for ST-HSCs and MPPs in central trained immunity. Conserved transcriptional and epigenetic alterations, including upregulation of C/EBPβ, increased chromatin accessibility at innate immunity genes marked by H3K4me3 (de Laval et al., 2020), and metabolic reprogramming toward enhanced glycolysis Mills et al., 2024 have been shown to occur in LT-HSCs that confer trained immunity in transplant models. These same transcriptional and epigenetic alterations are conserved in the ST-HSC and MPP compartments of recipients of trained LT-HSCs, suggesting that these cells may act as potent producers of innate immune memory effector cells. Following the induction of innate immune training with BCG vaccination or the fungal cell wall component β-glucan, ST-HSCs and MPPs exhibited enhanced expression of gene sets involved in DNA replication and cell division, as well as upregulation of several myeloid-lineage transcription factors at the expense of lymphoid-lineage TFs, indicating a skewing toward myelopoiesis (Kaufmann et al., 2018; Mitroulis et al., 2018). However, whether this expansion is autonomously driven by ST-HSCs and MPPs or is a result of upstream reprogramming of LT-HSCs remains an open question. Profiling the clonal dynamics of individual HSPC subsets after training stimuli may provide insight into which compartments are being transiently activated versus durably ‘trained’. Lineage tracing approaches, including inducible genetic barcoding systems or tamoxifen-inducible Cre drivers (Li et al., 2023), offer a promising avenue to directly test the origins of expanded progenitor subsets in response to innate immune training.

ST-HSC and MPP compartments have high proliferative capacity and fast differentiation dynamics compared to LT-HSCs, potentially making them better suited to respond to immediate immunological demands. Not only do they exhibit more pronounced transcriptional responses upon inflammatory stimulation than LT-HSCs, but they also have greater expansion capacity. It has been demonstrated that ST-HSCs and MPPs, particularly MPP3s, increase in number following BCG- (Kaufmann et al., 2018)**,** β-glucan- (Mitroulis et al., 2018), and heme-induced (Jentho et al., 2021) trained immunity, reflecting an expansion of the myeloid-biased progenitor pool. This expansion, coupled with conserved transcriptional changes, ensures a sustained supply of functionally reprogrammed myeloid cells that have been shown to execute robust antimicrobial activity against secondary challenge with *M. tb* (Kaufmann et al., 2018) or display enhanced metabolic activity in response to LPS (Mitroulis et al., 2018).

A key outstanding question in the field is whether LT-HSCs act as memory reservoirs that transmit trained states downstream to shorter-lived progenitors, or if ST-HSCs and MPPs are directly reprogrammed by inflammatory signals without input from LT-HSCs. In our work, we found that transplantation of sorted LT-HSCs alone was not sufficient to confer *in vivo* protection in an *M. avium* model of trained immunity (Kain et al., 2023), suggesting that ST-HSCs and MPPs rather than LT-HSCs are responsible for innate immune memory conservation. Nevertheless, further studies are required for confirmation. In addition, the degree to which each cell compartment contributes to innate memory conservation may depend on the nature, duration, and intensity of the training stimulus.

An indirect test of the functional capacities of ST-HSC- and MPP-derived innate effector cells is to derive monocytes and macrophages from whole bone marrow preparations. Through a 7-day derivation period, whole bone marrow is cultured with granulocyte–macrophage colony-stimulating factor to generate bone-marrow-derived macrophages (BMDMs). Given that ST-HSCs and MPPs are more abundant and produce more BMDMs in such culture conditions compared to LT-HSCs, BMDM cultures provide a useful way to assess how ST-HSCs and MPPs contribute to trained immunity. It has been shown that BMDMs cultured from whole bone marrow of BCG-vaccinated mice are epigenetically imprinted to initiate a more protective response against *M. tb* challenge (Kaufmann et al., 2018). The initial *in vivo* training is dependent on IFN-γ, as epigenetic reprogramming of hematopoietic progenitors in BCG-induced trained immunity models does not occur when the IFN-γ receptor is ablated (Mitroulis et al., 2018; Kaufmann et al., 2018). Several studies have indicated that IFN-γ is required in both establishment of trained immunity and T-cell activated bacterial control (Flynn et al., 1993; Akter et al., 2022).

In contrast, maladaptive induction of innate immune memory with *M. tb* has been shown to impede myelopoiesis via a type I IFN-dependent process (Khan et al., 2020). Similarly, a high-fat, high-calorie Western diet (Christ et al., 2018) reprograms ST-HSCs and MPPs via IL-1β, resulting in reduced MPP abundance and expanded lymphoid-biased MPP4 populations. These alterations result in impaired host immunity with chronic low-grade inflammation (Christ et al., 2018) and a weakened ability to control infection (Khan et al., 2020).

Collectively, these findings using non-transplant models highlight that ST-HSCs and MPPs may play a significant role in innate immune memory conservation and amplification.

### Innate immune memory conservation in granulocyte–monocyte progenitors

While the focus of central trained immunity research has been on HSCs and MPPs, strong evidence supports a downstream role of granulocyte–monocyte progenitors (GMPs) in central trained immunity. As mentioned above, central trained immunity can lead to a heterogeneity of responders and non-responders within the host (Verma et al., 2017; Kain et al., 2023), and cellular expansion can be limited to specific subsets of progenitors including MPP3s and GMPs (Kaufmann et al., 2018; Kalafati et al., 2020; Cheong et al., 2023; Christ et al., 2018; Kain et al., 2023; Arts et al., 2018).

As many studies highlight, significant expansion of GMPs occurs in response to training stimulants including β-glucan (Kalafati et al., 2020; Mitroulis et al., 2018), LPS (de Laval et al., 2020)**,** *M. avium* (Kain et al., 2023)**,** *M.* tb (Khan et al., 2020), polymicrobial sepsis (Bomans et al., 2018), and in autoimmune disease models (Mills et al., 2024). Transcriptional and epigenetic studies of GMPs have found a markedly pro-inflammatory signature in GMPs recovered from patients with long COVID-19 infection compared to healthy controls (Cheong et al., 2023). Additionally, β-glucan-trained GMPs, which were able to prevent melanoma growth, acquired epigenetic modifications that led to increased chromatin accessibility at genes related to neutrophil immunity, including ROS-producing factors *Ncf1* and *Ncf2* and type I interferon-related factors *Ifnar1*, *Irf1*, and *Ifitm1-3* (Kalafati et al., 2020). A study in patients who had recovered from SARS-CoV-2 infection similarly reported a strong type I interferon signature in GMPs both at the single-cell transcription level and chromatin accessibility level (Cheong et al., 2023). Indeed, both type I and type II interferons are known to promote HSC division and have been shown to be sufficient to induce central trained immunity by reprogramming HSPCs to result in increased killing capacity of BMDMs and long-lasting metabolic rewiring (Kain et al., 2023; Baldridge et al., 2010; Essers et al., 2009). Notably, GMPs are known to mount a strong response to interferons and, in one study, exhibited a higher level of IRF1 motif enrichment relative to HSCs and MPPs (Cheong et al., 2023), highlighting a potential strong contribution of GMPs toward the generation of central trained immunity. However, considering their short life span, it is unlikely that GMPs alone harbor the capacity to maintain long-term central trained immunity.

## Epigenetic mechanisms underlying central trained immunity

**Figure 3.**
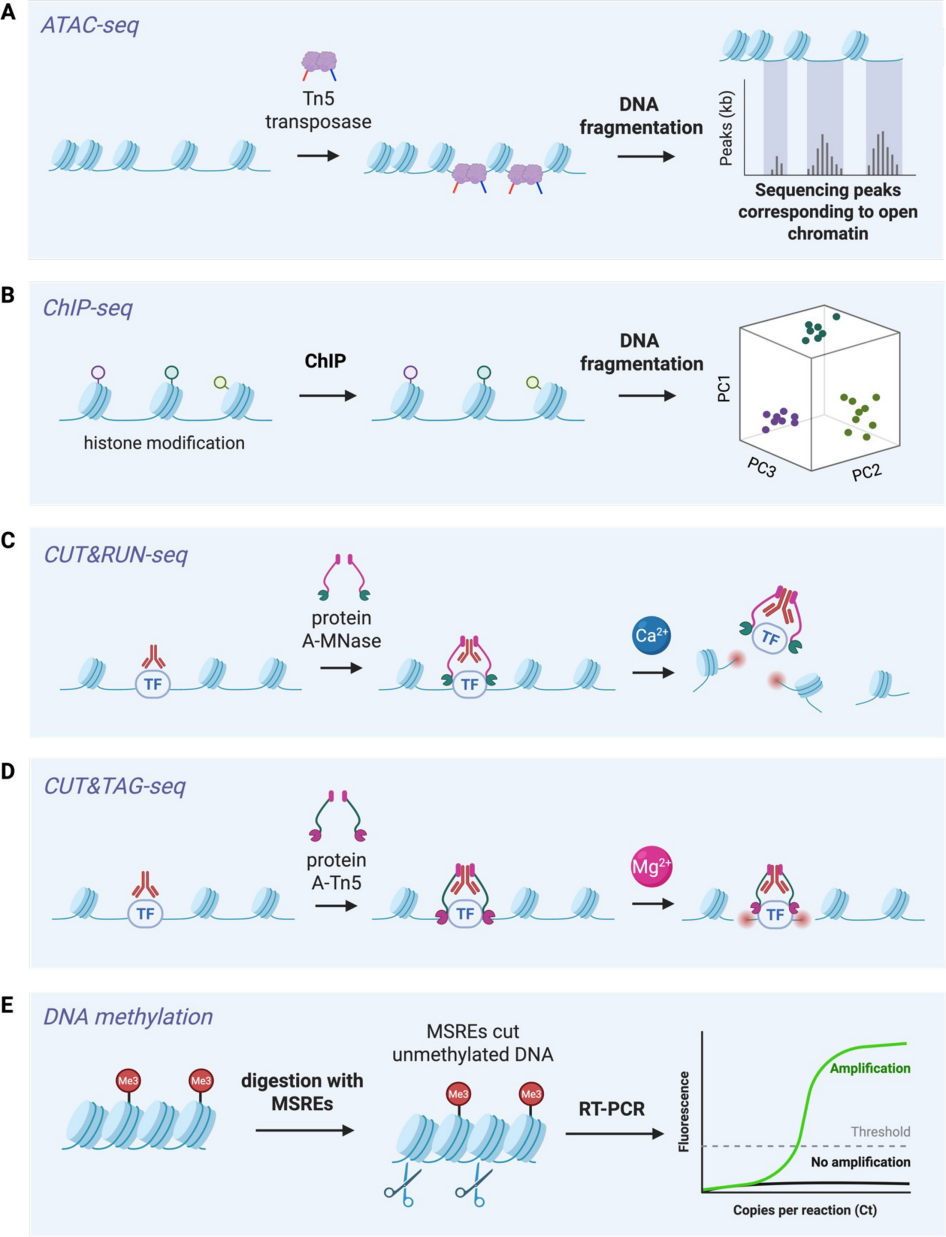
Methods for analysis of DNA epigenetic modifications. (**A**) ATAC-seq (Assay for Transposase-Accessible Chromatin with sequencing). This method determines chromatin accessibility by measuring open chromatin regions with Tn5 transposase, which inserts sequencing adapters into open chromatin regions. These regions are enriched in the sequencing results and correspond to larger sequencing peaks, reflecting where in the genome more transcriptional activity is occurring (Kaufmann et al., 2018; Cirovic et al., 2020; Mills et al., 2024; Cheong et al., 2023; Christ et al., 2018; Khan et al., 2020; de Laval et al., 2020; Kaufmann et al., 2022; Johansson et al., 2023; Sun et al., 2014; Zhang et al., 2017). (**B**) ChIP-seq (Chromatin Immunoprecipitation with sequencing). This method allows for the determination of protein-binding sites on DNA. Briefly, DNA sequences bound by transcription factors or histone modifications are captured via cross-linking, fragmentation, and immunoprecipitation using an antibody specific to the protein of interest. This method provides data in the form of genome-wide maps illustrating areas of histone modifications or transcription factor binding. (**C**) CUT&RUN-seq (Cleavage Under Targets and Release Using Nuclease sequencing). Much like ChIP-seq, CUT&RUN-seq provides genome-wide maps of protein–DNA interactions, but has a higher resolution and lower background. This method uses an antibody to bind a target protein, such as a transcription factor, followed by recruitment of micrococcal nuclease (A-MNase) fusion protein. This cleaves DNA only near the antibody-bound region, causing release of those fragments for sequencing (Ochando et al., 2023; Le et al., 2023). (**D**) CUT&TAG-seq (Cleavage Under Targets and TAGmentation sequencing). This method functions similarly to CUT&RUN-seq but provides data with even greater sensitivity and reduced background. After binding by an antibody to the protein of interest, a fusion of protein A to Tn5 transposase directly cuts and inserts sequencing adapters into the DNA at the DNA–protein-binding sites. (**E**) DNA methylation. Genomic DNA is digested with methylation-sensitive restriction enzymes (MSREs), cleaving unmethylated DNA while leaving methylated DNA intact. Digested DNA is then analyzed by reverse transcription-polymerase chain reaction (RT-PCR) with primers that flank the region of interest. Only methylated DNA is amplified, allowing for methylation to be determined by comparing the copies per reaction (Ct) between MSRE-treated and control samples (Verma et al., 2017; Mitroulis et al., 2018; Kain et al., 2023; Sun et al., 2024; Hormaechea-Agulla et al., 2021; Johansson et al., 2023).

Mechanistically, inflammation has been shown to correlate with chromatin modifications in HSPCs that are passed on to downstream cells to amplify subsequent responses to inflammatory challenges (Netea et al., 2020; Cirovic et al., 2020). Both generation and maintenance of innate immune memory are associated with chromatin modifications such as methylation, lactylation, and acetylation of histones that alter chromatin accessibility and poise specific DNA loci for faster, higher transcriptional induction (Netea et al., 2020; Ochando et al., 2023, Ziogas et al., 2025). Many next-generation sequencing techniques, such as low-input chromatin immunoprecipitation sequencing (ChIP-seq), assay for transposase accessible chromatin (ATAC-seq), cleavage under targets and release using nuclease (CUT&RUN-seq), and cleavage under targets and tagmentation (CUT&TAG-seq), and single-cell sequencing techniques, have permitted researchers to determine the chromatin landscape in these HSPC subpopulations (Ochando et al., 2023; Figure 3).

In mice, studies have shown that activating histone modifications like H3K4me3 and H3K27ac are enriched at genes associated with innate immune memory such as the metabolic regulator mTOR (Arts et al., 2016; Domínguez-Andrés et al., 2019) and pro-inflammatory cytokines IL-6 and TNF-α (Arts et al., 2016; Saeed et al., 2014). Further, sorted HSPCs (LSK+, Flt3-) have been observed to gain H3K4 mono- and tri-methylation and increased chromatin accessibility in response to LPS stimulation at genes involved in inflammatory biological processes, including innate immune response, myeloid differentiation, and anti-bacterial responses (de Laval et al., 2020). Notably, upon secondary stimulation, these LPS-trained HSPCs demonstrate a heightened excitability and a significant gain of activating histone marks H3K4me1, H3K27ac, and an overall increase in chromatin accessibility at loci associated with stem cell activation (de Laval et al., 2020). Using CUT&RUN sequencing, activating H3K27ac marks in SLAM-HSCs (Lin-, cKit+, CD48−, CD150+) have been shown to correlate with enhanced cytokine production such as the chemokine CCL5, upon *M. avium* infection (Le et al., 2023). Finally, metabolic processes, like oxidative phosphorylation (Sun et al., 2024) and glycolysis (Mills et al., 2024), coincide with immune training (Abhimanyu et al., 2024). These findings highlight the potential for HSCs to gain common activating histone modifications upon inflammatory stimulation, contributing to trained immunity phenotypes. However, more studies into the histone landscape of HSPCs upon inflammation are needed, as a wide range of different modifications and their mechanistic contribution to the memory phenotype remain unexplored.

Single-cell sequencing studies in human HSPCs corroborate that long-term transcriptional and epigenetic reprogramming in HSPCs can be retained in myeloid-lineage cells. For example, BCG-trained CD34^+^ human HSPCs retained transcriptional bias and chromatin accessibility toward neutrophil activation (Cirovic et al., 2020; Sun et al., 2024). While this reprogramming did not alter cell numbers or baseline function, these BCG-trained bone marrow-derived mononuclear cells showed heightened cytokine responses after being challenged with *Candida albicans*. Furthermore, a recent study showed that inflammation-induced reprogramming of HSCs and GMPs could be detected in human patients 6 months after recovery from SARS-CoV-2 infection, resulting in a persistent myeloid differentiation bias and hyperinflammatory state (Cheong et al., 2023). These long-lasting changes included durable alterations in chromatin accessibility at genes such as *CXCR4*, *CCL5*, and *GBP5* that remained open for almost a year post-SARS-CoV-2 infection.

In addition to chromatin accessibility and histone modifications, DNA methylation has also been found to be altered in HSPCs after infection. DNA methyltransferase DNMT3A is the main cellular enzyme responsible for de novo methylation of cytosine to generate 5-methylcytosine, a mark that typically represses transcription. Interestingly, we and others showed that loci encoding transcription factors such as *Batf2*, *Fos*, and *Jun*, important in myeloid differentiation (Matatall et al., 2016; Liebermann et al., 1998; Taylor et al., 2024) were hypermethylated and transcriptionally repressed in *Dnmt3a*-mutant HSCs after infection (Hormaechea-Agulla et al., 2021). On the other hand, DNA methylation silences critical immune response genes in contexts like LPS or *M. tb*-induced immune tolerance (Abhimanyu et al., 2024). These findings imply that DNA methylation regulates gene transcription in the setting of infection. Despite these discoveries, the role of DNA methylation in central trained immunity remains largely unexplored.

A common set of transcription factor motifs has emerged across epigenetic studies of trained immunity. As noted above, we found that BATF2 was differentially regulated in response to infection in Dnmt3a-KO mice. Further, the loss of *Batf2* (Le et al., 2023) disrupted downstream macrophage responses to pathogen challenge, highlighting its role in initiation of immune responses (Le et al., 2023). Likewise, the transcription factor CEBPβ, which is known to open chromatin and promote transcription of other TFs (AP1, IRF, and ATF) as well as innate immune response and myeloid differentiation genes such as *Ifnar2*, *Mx2*, and *Itih5* (Matatall et al., 2016), has been shown to play a critical role in LPS-induced epigenetic reprogramming and central trained memory (de Laval et al., 2020). In patients with long-SARS-CoV-2, similar transcription factor programs were altered in HSPCs and monocytes, with heightened activity of TFs like JUN, NFKB1, and NFKB2, and interferon regulatory family TFs (Cheong et al., 2023). In embryonic epidermal stem cells during skin injury and repair, transcription factors including FOS and JUN act as a switch triggered by the initial training stimulus to direct stem cells’ response to different pathogens (Yang et al., 2023; Missinato et al., 2023). Altogether, these studies highlight the activation of specific TFs as a critical step in central trained immunity. This raises the possibility that transcription factors act as pioneer factors which open chromatin to enable the acquisition of DNA methylation and histone modifications. Whether chromatin marks or transcription factors are the key initiating event for central trained immunity remains an active area of research in the field (Netea et al., 2020; Ochando et al., 2023).

Altogether, histone modifications and chromatin openness at key transcription factor binding motifs have been shown to be conserved in HSCs and downstream effector myeloid cells after immune training, suggesting that conserved chromatin accessibility changes may provide the mechanism for enhanced innate immune responses in central trained immunity. However, the mechanisms by which such marks are passed down through ontogeny remain undefined. Are transcription factors stable enough to remain associated with chromatin through cell division and thereby facilitate histone modifications? Or are these histone modifications conserved through cell divisions that occur during differentiation? These challenging questions will serve as guideposts for future research.

## Heterogeneity of HSPC responses

While a variety of stimulants produce trained immunity phenotypes, a common set of inflammatory cytokines plays key mechanistic roles downstream of these stimulants. Cytokines including IL-6 (Cheong et al., 2023), IFNs (Kaufmann et al., 2018; Khan et al., 2020), and IL-1 (Moorlag et al., 2020) are commonly reported inducers of innate memory in models of trained immunity. These cytokines, particularly IL-6 and IL-1, can be produced both by HSPCs themselves (Granick et al., 2012), their progeny, as well as immune and niche cells in the bone marrow microenvironment which therefore contribute significantly to trained immunity induction (Mitroulis et al., 2020). We demonstrated that IFNγ is essential for trained immunity, as the loss of the IFNγ receptor resulted in the abrogation of trained immunity protection (Kaufmann et al., 2018). IL-1 and type I IFN are crucial for β-glucan-mediated trained immunity (Kalafati et al., 2020; Mitroulis et al., 2018; Moorlag et al., 2020; Gow et al., 2007)**,** leading to proliferation and differential skewing of hematopoietic progenitor cells. Specifically, GMPs, Gr1^+^CD11b^+^ cells, and myeloid cells proliferate and serve in a protective role, such as in neutrophil-mediated anti-cancer responses (Kalafati et al., 2020; Geller et al., 2022) and macrophage-mediated responses to control *M. tb* (Moorlag et al., 2020). Moorlag et al. demonstrated that reprogramming of HSPCs relied on IL-1, as inhibition of IL-1 signaling impaired the effects of β-glucan-mediated trained immunity (Moorlag et al., 2020).

These studies highlight the necessity of inflammatory cytokines for the generation of central trained immunity. Notably, inflammatory cytokines like IL-6 are elevated in aging and promote myeloid-biased differentiation and hyperinflammatory innate immune cells, a concept known as inflammaging (Chung et al., 2006). Considering the similarities between inflammaging and trained immunity, the question of whether cytokines alone can confer trained immunity is a field of active investigation. We showed that a single dose of recombinant IFNγ reprograms HSPCs leading to higher metabolism and more robust and efficient immune responses in the downstream macrophages (Kain et al., 2023). This phenotype persisted even after HSPC transplant. The question remaining is whether a strong enough cytokine signal, or combination of cytokines, is indeed sufficient to generate central trained immunity, or whether stimuli recognition itself has shaping potential for the epigenetic reprogramming and downstream immune response. Upcoming studies are geared to clarify whether ‘clean’ training stimulants, such as pure cytokine cocktails, are sufficient to promote central training and host immunity.

Notably, studies indicate that HSPCs do not all respond to inflammatory cytokine stimulation alike, opening the possibility that specific subpopulations get differentially programmed with every inflammatory insult. HSCs express receptors for inflammatory cytokines and pathogen-associated molecular patterns that shape HSC cellular and transcriptional outputs. Both human and murine HSPCs express many pathogen recognition receptors such as TLR-1, TLR2, TLR3, TLR4, TLR6, TLR7, TLR8, NOD, RigI-like receptors, and STING/GAS that can directly interact with stimuli, activate downstream signaling pathways, and promote HSC loss of quiescence (King and Goodell, 2011; Baldridge et al., 2010). For instance, HSCs process LPS through interactions with TLR4, recruiting C/EBPβ to result in a transient increase of LT-HSCs, MPP2, MPP3, and GMP numbers and stable changes in chromatin accessibility of Flt3^−^LSK cells (de Laval et al., 2020). Single-cell studies indicate that HSPCs differentially process inflammatory signals to initiate the reprogramming that produces and maintains central trained immunity, leading to heterogeneity in responses (Kain et al., 2023).

Despite the recognition that HSPCs are highly heterogeneous, it remains unknown if all HSPCs have equal capacity for innate immune memory. Initial studies of BCG-vaccinated humans found that not all patients developed trained immune memory (Verma et al., 2017). Taking peripheral monocytes from healthy volunteers pre and post BCG vaccination, Verma et al. described varying levels of cytokine production in response to *in vitro* stimulation, thus categorizing subjects into ‘responders’ and ‘non-responders’. BCG responsive changes were attributed to DNA hypomethylation at regions such as *ADCY3*, leading to immune activation. The researchers did not study histone modifications in this study, but others have recently found increased chromatin accessibility in HSPCs of BCG-vaccinated individuals (Sun et al., 2024). This variability in response raises questions about the universality of trained immunity as an immunization strategy and highlights that genetic factors or environmental conditions may contribute to trained immunity in yet unknown ways.

Responder and non-responder variability has also been observed on a cellular level in HSPC single-cell transcriptomic studies in various inflammation models, including BCG vaccination (Kaufmann et al., 2018), LPS stimulation (de Laval et al., 2020), and *M. avium* infection (Kain et al., 2023). In some cases, distinct transcriptional states within HSPCs could be clustered in alignment with fate determination. Specifically, single-cell RNA sequencing on bone marrow HSPCs revealed multiple clusters of MPP3s, with one cluster containing a transcriptional signature indicative of granulocytic bias (Kaufmann et al., 2022). Similarly, a single-cell RNA sequencing study of HSPCs in the setting of *M. avium* infection revealed heterogenous transcriptional responses, with a minority of cells driving the significant upregulation of inflammatory genes (Kain et al., 2023). Altogether, these single-cell studies indicate that HSPC populations do not respond as a monolith to inflammatory stimuli; rather, there is significant heterogeneity even within cell types to produce appropriate inflammatory responses. Thus, trained immunity phenotypes may be driven by a small subset of total HSPCs. It is tempting to speculate that heterogeneous reprogramming is an adaptive feature, allowing non-responsive HSPCs to continue basal functions or be poised for future training by alternative agents. Alternatively, heterogeneity may be due to spatial variation in infection and inflammatory cytokine density and spatial distribution of the HSPCs in the bone marrow. Spatial transcriptomics methods (Crosse et al., 2020) could shed light on whether ‘responder’ cells are clustered relative to hubs of infection or mature responder cells producing cytokines in the bone marrow.

With the expansion of single-cell sequencing, a growing body of literature suggests heterogeneity even within the canonically defined HSC compartment (LSK, CD150-negative, CD48-positive) at baseline and upon inflammatory activation. For instance, myeloid-biased CD41-positive HSCs (Gekas and Graf, 2013) are a potential driver of central trained immunity (Mitroulis et al., 2018; Kain et al., 2023; de Laval et al., 2020). We recently described an infection-activated HSC subset (IA-HSC) stimulated by *M. avium* that expresses both LSK and activation markers, such as CD81, CD24a, and CD9 (Kain et al., 2023). Notably, the IA-HSCs can be sorted and transplanted in murine models, thus providing the potential to investigate whether these cells are the source of trained immunity. Similar subsets of ‘inflammatory memory’ HSCs have been identified in human studies (Zeng et al., 2023). Future studies aim at parsing out how the heterogeneity in HSCs relates to trained immunity phenotypes, while deeper epigenetic sequencing methodologies (Figure 3) will enable further definition of HSC subpopulations both in health and disease in humans (Zeng et al., 2023).

Preferential expansion of a specific HSPC subset is reminiscent of clonal hematopoiesis of indeterminate potential (CHIP), a preleukemic condition in which the bone marrow of older individuals harbors an expanding population of cell clones with somatic mutations, often in cancer-driver genes (Jaiswal and Ebert, 2019). Like trained immunity, CHIP clones can respond to environmental stressors like inflammation and significantly expand their progeny, ultimately gaining a cellular advantage in the bone marrow and peripheral blood (Cao et al., 2024; Florez et al., 2022; Avagyan and Zon, 2023). Inflammation-mediated expansion, epigenetic changes, and cancer predisposition are fundamental themes shared between the fields of central trained immunity and CHIP (Chavakis et al., 2022). As such, it is worthwhile to consider whether responder cells poised for trained immunity exist within hosts in the same way that rare somatic mutations arise, and whether inflammatory training may lead to their expansion and selective advantage like in CHIP clones. Indeed, if innate immune memory is actually achieved by selection and expansion of preexisting populations, perhaps central trained immunity is really a manifestation of clonal selection rather than training and immunological memory.

## Summary and future directions

In sum, recent studies provide strong evidence that HSPCs can cell autonomously sustain trained immunity phenotypes in both mice and humans, supporting long-term production of innate memory effector cells with enhanced immune function. The mechanisms by which HSPCs support central trained immunity are fascinating. Here, we have described two major and non-mutually exclusive mechanisms: (1) inflammatory stimuli select and alter lineage fate determination of HSPC progeny, reshaping immune cell populations; (2) reprogramming of HSPCs generates downstream progeny that are transcriptionally and metabolically poised for enhanced immune function. Indeed, both mechanisms seem to occur in parallel. While the field of central trained immunity continues to expand, many unknowns remain. The following questions remain critical priorities to translate central trained immunity for therapeutic benefit.

First, epigenetic mechanisms by which HSPCs maintain central trained immunity remain poorly understood. Transplant studies indicate that HSPCs sustain changes that last for months or years and can be passed to progeny cells, implying an epigenetic change. While histone modifications and DNA methylation are considered the mediators of reprogramming signatures, the causal relationship between chromatin marks and trained immunity has not been defined through loss of function studies. Likewise, the association of specific epigenetic marks with defined training results is still pending. Most studies have focused on H3K4me3 and H3K27ac, but these are typically transient marks; a number of other marks may be better candidates for memory functions. Loss and gain of function studies will indisputably demonstrate a role for a particular chromatin modification, gene locus, or transcription factor in trained immunity, which can ultimately also be therapeutically targeted for enhancement or clearance.

Second, alternative mechanisms for HSC memory induction such as sustained changes in mitochondria need to be considered (Bonora et al., 2024; Hinge et al., 2020). Just as epigenetic reprogramming is critical for trained immunity, it is well documented that metabolic rewiring, particularly at the mitochondrial level, is critical for innate immune memory (Netea et al., 2020; Arts et al., 2016; Abhimanyu et al., 2024). Notably, the methyl groups and acetyl-CoA that serve as key substrates for epigenetic modifications are products of mitochondrial metabolism (Netea et al., 2020; Ferreira et al., 2024; Riksen and Netea, 2021; Wu et al., 2023). Considering that alterations in critical metabolic pathways such as oxidative phosphorylation in HSPCs are consistently observed in trained immunity models (Mills et al., 2024; Sun et al., 2024; Abhimanyu et al., 2024), metabolic rewiring may in fact be the driver of epigenetic alterations.

Third, as described throughout this review, hematopoietic cells at different stages of differentiation, from HSCs to lineage-committed progenitors, contribute to central trained immunity. The HSPC subcompartment most critical for trained immunity remains unknown, and the major responders and mediators may be context dependent. Current research relies on transplantation of mixed HSPC populations that are limited by the low number of primitive cells and short lifespan of downstream progeny. It is worth acknowledging that murine transplant studies do not rule out the possibility that trained immunity may be transmitted by non-cell autonomous mechanisms (e.g. alterations of the niche). However, they do provide strong rationale for pursuing further mechanistic studies of cell autonomous central trained immunity, with potential benefits for HSC transplant recipients. In the future, *in vivo* barcoding and mosaic models will enhance these studies. Single-cell and spatial multiomic studies will ultimately address how many reprogrammed HSPCs are necessary to generate functionally relevant degrees of immune protection. Furthermore, these studies will elucidate whether innate memory protection induced by one specific stimulus diminishes trained immunity potential for other stimuli. Finally, detailed studies of changes in the bone marrow niche after inflammatory challenge will shed light on non-cell autonomous mechanisms for central trained immunity.

Enhanced immunity conferred by innate immune training has exciting translational potential. Providing nonspecific enhanced immunity would be of critical value in the setting of novel pandemics for which antigen-specific vaccines are not yet available. Further, immune training of HSPCs could improve outcomes for recipients of bone marrow transplants, for whom infection remains a leading cause of death. However, wide heterogeneity in HSPC transcriptional responses and epigenetic marks in trained immunity studies, in both murine and human trials, remains a major limiting factor for translation of trained immunity. Studies such as those published by Verma et al. show that current approaches to induce trained immunity may not be successful for everyone, even with a healthy background (Verma et al., 2017). Furthermore, the variable memory observed in long-COVID patients indicates that trained immunity responses may be maladaptive for some individuals (Cheong et al., 2023). Thus, many more studies to explicate the cellular and molecular basis of trained immunity are needed to actualize these goals.

In summary, it is an exciting time for research into central trained immunity. Inflammatory training of HSPCs confers long-term enhanced immunity against secondary infectious challenges with measurable changes in prevalence and function of progenitors and innate immune cells. Current studies indicate that immunological training throughout life may shape the evolution of the stem and progenitor cell compartment, yielding manifold layers of immune memory. This phenomenon has been described in human epidemiologic and single-cell functional studies and is reproducible in mouse models, with clear implications for human health. While the crucial role of HSPCs in maintenance of memory function in innate immune cells has been revealed, the field is now poised to address the detailed mechanistic basis for this phenomenon. Future clinical research envisions the development of methodologies to train donor HSPCs prior to transplant, resulting in reduced infectious complications after transplant. Alternatively, elucidation of cellular sources of trained immunity may lead to strategies to select for these cells as an immunologically enhanced donor pool. Finally, elucidating the epigenetic bases for trained immunity may lead to bioengineering approaches that recreate these effects in patients without the need for immune training. Such approaches can be utilized in the clinic to offset immune dysfunction in aging, to enhance tumor immunotherapy, or to curb the dangerous effects of autoimmunity.

Table 1 describes the different subpopulations of HSPCs that have been shown to conserve trained immunity signatures. Depending on the HSPC population, trained immunity has still been observed at specific selected experimental endpoints spanning from a few weeks to years post-training. Reversal or loss of training signatures has yet to be demonstrated.

## References

[bib1] Abhimanyu A, Longlax SC, Nishiguchi T, Ladki M, Sheikh D, Martinez AL, Mace EM, Grimm SL, Caldwell T, Portillo Varela A, Sekhar RV, Mandalakas AM, Mlotshwa M, Ginidza S, Cirillo JD, Wallis RS, Netea MG, van Crevel R, Coarfa C, DiNardo AR (2024). TCA metabolism regulates DNA hypermethylation in LPS and *Mycobacterium tuberculosis*-induced immune tolerance. PNAS.

[bib2] Akter S, Chauhan KS, Dunlap MD, Choreño-Parra JA, Lu L, Esaulova E, Zúñiga J, Artyomov MN, Kaushal D, Khader SA (2022). *Mycobacterium tuberculosis* infection drives a type I IFN signature in lung lymphocytes. Cell Reports.

[bib3] Al-Amoodi AS, Li Y, Al-Ghuneim A, Allehaibi H, Isaioglou I, Esau LE, AbuSamra DB, Merzaban JS (2022). Refining the migration and engraftment of short-term and long-term HSCs by enhancing homing-specific adhesion mechanisms. Blood Advances.

[bib4] Anjos-Afonso F, Bonnet D (2023). Human CD34+ hematopoietic stem cell hierarchy: how far are we with its delineation at the most primitive level?. Blood.

[bib5] Arts RJW, Carvalho A, La Rocca C, Palma C, Rodrigues F, Silvestre R, Kleinnijenhuis J, Lachmandas E, Gonçalves LG, Belinha A, Cunha C, Oosting M, Joosten LAB, Matarese G, van Crevel R, Netea MG (2016). Immunometabolic pathways in BCG-induced trained immunity. Cell Reports.

[bib6] Arts RJW, Moorlag S, Novakovic B, Li Y, Wang SY, Oosting M, Kumar V, Xavier RJ, Wijmenga C, Joosten LAB, Reusken C, Benn CS, Aaby P, Koopmans MP, Stunnenberg HG, van Crevel R, Netea MG (2018). BCG Vaccination protects against experimental viral infection in humans through the induction of cytokines associated with trained immunity. Cell Host & Microbe.

[bib7] Avagyan S, Zon LI (2023). Clonal hematopoiesis and inflammation - the perpetual cycle. Trends in Cell Biology.

[bib8] Baldridge MT, King KY, Boles NC, Weksberg DC, Goodell MA (2010). Quiescent haematopoietic stem cells are activated by IFN-gamma in response to chronic infection. Nature.

[bib9] Belle JI, Petrov JC, Langlais D, Robert F, Cencic R, Shen S, Pelletier J, Gros P, Nijnik A (2016). Repression of p53-target gene Bbc3/PUMA by MYSM1 is essential for the survival of hematopoietic multipotent progenitors and contributes to stem cell maintenance. Cell Death and Differentiation.

[bib10] Bloom M, Malouf C, Rodriguez-Fraticelli A, Wilkinson AC, Sankaran VG, Cvejic A (2023). Exploiting somatic mutations to decipher human blood production: a natural lineage-tracing strategy. Experimental Hematology.

[bib11] Bomans K, Schenz J, Sztwiertnia I, Schaack D, Weigand MA, Uhle F (2018). Sepsis induces a long-lasting state of trained immunity in bone marrow monocytes. Frontiers in Immunology.

[bib12] Bonora M, Morganti C, van Gastel N, Ito K, Calura E, Zanolla I, Ferroni L, Zhang Y, Jung Y, Sales G, Martini P, Nakamura T, Lasorsa FM, Finkel T, Lin CP, Zavan B, Pinton P, Georgakoudi I, Romualdi C, Scadden DT, Ito K (2024). A mitochondrial NADPH-cholesterol axis regulates extracellular vesicle biogenesis to support hematopoietic stem cell fate. Cell Stem Cell.

[bib13] Busch K, Rodewald HR (2016). Unperturbed vs. post-transplantation hematopoiesis: both in vivo but different. Current Opinion in Hematology.

[bib14] Cabezas-Wallscheid N, Klimmeck D, Hansson J, Lipka DB, Reyes A, Wang Q, Weichenhan D, Lier A, von Paleske L, Renders S, Wünsche P, Zeisberger P, Brocks D, Gu L, Herrmann C, Haas S, Essers MAG, Brors B, Eils R, Huber W, Milsom MD, Plass C, Krijgsveld J, Trumpp A (2014). Identification of regulatory networks in HSCs and their immediate progeny via integrated proteome, transcriptome, and DNA methylome analysis. Cell Stem Cell.

[bib15] Cao R, Thatavarty A, King KY (2024). Forged in the fire: Lasting impacts of inflammation on hematopoietic progenitors. Experimental Hematology.

[bib16] Challen GA, Boles N, Lin KKY, Goodell MA (2009). Mouse hematopoietic stem cell identification and analysis. Cytometry. Part A.

[bib17] Challen GA, Pietras EM, Wallscheid NC, Signer RAJ (2021). Simplified murine multipotent progenitor isolation scheme: Establishing a consensus approach for multipotent progenitor identification. Experimental Hematology.

[bib18] Chanut FJA, Sanvito F, Ferrari G, Visigalli I, Carriglio N, Hernandez RJ, Norata R, Doglioni C, Naldini L, Cristofori P (2021). Conditioning regimens in long-term pre-clinical studies to support development of ex vivo gene therapy: review of nonproliferative and proliferative changes. Human Gene Therapy.

[bib19] Chavakis T, Wielockx B, Hajishengallis G (2022). Inflammatory modulation of hematopoiesis: linking trained immunity and clonal hematopoiesis with chronic disorders. Annual Review of Physiology.

[bib20] Cheng SC, Quintin J, Cramer RA, Shepardson KM, Saeed S, Kumar V, Giamarellos-Bourboulis EJ, Martens JHA, Rao NA, Aghajanirefah A, Manjeri GR, Li Y, Ifrim DC, Arts RJW, van der Veer B, Deen PMT, Logie C, O’Neill LA, Willems P, van de Veerdonk FL, van der Meer JWM, Ng A, Joosten LAB, Wijmenga C, Stunnenberg HG, Xavier RJ, Netea MG (2014). mTOR- and HIF-1α-mediated aerobic glycolysis as metabolic basis for trained immunity. Science.

[bib21] Cheng H, Zheng Z, Cheng T (2020). New paradigms on hematopoietic stem cell differentiation. Protein & Cell.

[bib22] Cheong JG, Ravishankar A, Sharma S, Parkhurst CN, Grassmann SA, Wingert CK, Laurent P, Ma S, Paddock L, Miranda IC, Karakaslar EO, Nehar-Belaid D, Thibodeau A, Bale MJ, Kartha VK, Yee JK, Mays MY, Jiang C, Daman AW, Martinez de Paz A, Ahimovic D, Ramos V, Lercher A, Nielsen E, Alvarez-Mulett S, Zheng L, Earl A, Yallowitz A, Robbins L, LaFond E, Weidman KL, Racine-Brzostek S, Yang HS, Price DR, Leyre L, Rendeiro AF, Ravichandran H, Kim J, Borczuk AC, Rice CM, Jones RB, Schenck EJ, Kaner RJ, Chadburn A, Zhao Z, Pascual V, Elemento O, Schwartz RE, Buenrostro JD, Niec RE, Barrat FJ, Lief L, Sun JC, Ucar D, Josefowicz SZ (2023). Epigenetic memory of coronavirus infection in innate immune cells and their progenitors. Cell.

[bib23] Christ A, Günther P, Lauterbach MAR, Duewell P, Biswas D, Pelka K, Scholz CJ, Oosting M, Haendler K, Baßler K, Klee K, Schulte-Schrepping J, Ulas T, Moorlag SJCFM, Kumar V, Park MH, Joosten LAB, Groh LA, Riksen NP, Espevik T, Schlitzer A, Li Y, Fitzgerald ML, Netea MG, Schultze JL, Latz E (2018). Western diet triggers nlrp3-dependent innate immune reprogramming. Cell.

[bib24] Chung HY, Sung B, Jung KJ, Zou Y, Yu BP (2006). The molecular inflammatory process in aging. Antioxidants & Redox Signaling.

[bib25] Ciarlo E, Heinonen T, Théroude C, Asgari F, Le Roy D, Netea MG, Roger T (2020). Trained immunity confers broad-spectrum protection against bacterial infections. The Journal of Infectious Diseases.

[bib26] Cimato TR, Furlage RL, Conway A, Wallace PK (2016). Simultaneous measurement of human hematopoietic stem and progenitor cells in blood using multicolor flow cytometry. Cytometry. Part B, Clinical Cytometry.

[bib27] Cirovic B, de Bree LCJ, Groh L, Blok BA, Chan J, van der Velden W, Bremmers MEJ, van Crevel R, Händler K, Picelli S, Schulte-Schrepping J, Klee K, Oosting M, Koeken V, van Ingen J, Li Y, Benn CS, Schultze JL, Joosten LAB, Curtis N, Netea MG, Schlitzer A (2020). BCG vaccination in humans elicits trained immunity via the hematopoietic progenitor compartment. Cell Host & Microbe.

[bib28] Crosse EI, Gordon-Keylock S, Rybtsov S, Binagui-Casas A, Felchle H, Nnadi NC, Kirschner K, Chandra T, Tamagno S, Webb DJ, Rossi F, Anderson RA, Medvinsky A (2020). Multi-layered Spatial transcriptomics identify secretory factors promoting human hematopoietic stem cell development. Cell Stem Cell.

[bib29] Cunningham KT, Finlay CM, Mills KHG (2021). Helminth imprinting of hematopoietic stem cells sustains anti-inflammatory trained innate immunity that attenuates autoimmune disease. Journal of Immunology.

[bib30] de Laval B, Maurizio J, Kandalla PK, Brisou G, Simonnet L, Huber C, Gimenez G, Matcovitch-Natan O, Reinhardt S, David E, Mildner A, Leutz A, Nadel B, Bordi C, Amit I, Sarrazin S, Sieweke MH (2020). C/EBPβ-Dependent epigenetic memory induces trained immunity in hematopoietic stem cells. Cell Stem Cell.

[bib31] Ding Y, Liu Z, Liu F (2021). Transcriptional and epigenetic control of hematopoietic stem cell fate decisions in vertebrates. Developmental Biology.

[bib32] Domínguez-Andrés J, Novakovic B, Li Y, Scicluna BP, Gresnigt MS, Arts RJW, Oosting M, Moorlag S, Groh LA, Zwaag J, Koch RM, Ter Horst R, Joosten LAB, Wijmenga C, Michelucci A, van der Poll T, Kox M, Pickkers P, Kumar V, Stunnenberg H, Netea MG (2019). The itaconate pathway is a central regulatory node linking innate immune tolerance and trained immunity. Cell Metabolism.

[bib33] Ema H, Morita Y, Suda T (2014). Heterogeneity and hierarchy of hematopoietic stem cells. Experimental Hematology.

[bib34] Essers MAG, Offner S, Blanco-Bose WE, Waibler Z, Kalinke U, Duchosal MA, Trumpp A (2009). IFNalpha activates dormant haematopoietic stem cells in vivo. Nature.

[bib35] Ferreira AV, Domínguez-Andrés J, Merlo Pich LM, Joosten LAB, Netea MG (2024). Metabolic regulation in the induction of trained immunity. Seminars in Immunopathology.

[bib36] Florez MA, Tran BT, Wathan TK, DeGregori J, Pietras EM, King KY (2022). Clonal hematopoiesis: Mutation-specific adaptation to environmental change. Cell Stem Cell.

[bib37] Flynn JL, Chan J, Triebold KJ, Dalton DK, Stewart TA, Bloom BR (1993). An essential role for interferon gamma in resistance to *Mycobacterium tuberculosis* infection. The Journal of Experimental Medicine.

[bib38] Gekas C, Graf T (2013). CD41 expression marks myeloid-biased adult hematopoietic stem cells and increases with age. Blood.

[bib39] Geller AE, Shrestha R, Woeste MR, Guo H, Hu X, Ding C, Andreeva K, Chariker JH, Zhou M, Tieri D, Watson CT, Mitchell RA, Zhang H, Li Y, Martin II RCG, Rouchka EC, Yan J (2022). The induction of peripheral trained immunity in the pancreas incites anti-tumor activity to control pancreatic cancer progression. Nature Communications.

[bib40] Gow NAR, Netea MG, Munro CA, Ferwerda G, Bates S, Mora-Montes HM, Walker L, Jansen T, Jacobs L, Tsoni V, Brown GD, Odds FC, Van der Meer JWM, Brown AJP, Kullberg BJ (2007). Immune recognition of Candida albicans beta-glucan by dectin-1. The Journal of Infectious Diseases.

[bib41] Granick JL, Simon SI, Borjesson DL (2012). Hematopoietic stem and progenitor cells as effectors in innate immunity. Bone Marrow Research.

[bib42] Hasan T, Pasala AR, Hassan D, Hanotaux J, Allan DS, Maganti HB (2024). Homing and engraftment of hematopoietic stem cells following transplantation: a pre-clinical perspective. Current Oncology.

[bib43] Hernández-Malmierca P, Vonficht D, Schnell A, Uckelmann HJ, Bollhagen A, Mahmoud MAA, Landua SL, van der Salm E, Trautmann CL, Raffel S, Grünschläger F, Lutz R, Ghosh M, Renders S, Correia N, Donato E, Dixon KO, Hirche C, Andresen C, Robens C, Werner PS, Boch T, Eisel D, Osen W, Pilz F, Przybylla A, Klein C, Buchholz F, Milsom MD, Essers MAG, Eichmüller SB, Hofmann WK, Nowak D, Hübschmann D, Hundemer M, Thiede C, Bullinger L, Müller-Tidow C, Armstrong SA, Trumpp A, Kuchroo VK, Haas S (2022). Antigen presentation safeguards the integrity of the hematopoietic stem cell pool. Cell Stem Cell.

[bib44] Hersh EM, Gutterman JU, Mavligit GM (1977). BCG as adjuvant immunotherapy for neoplasia. Annual Review of Medicine.

[bib45] Hinge A, He J, Bartram J, Javier J, Xu J, Fjellman E, Sesaki H, Li T, Yu J, Wunderlich M, Mulloy J, Kofron M, Salomonis N, Grimes HL, Filippi MD (2020). Asymmetrically segregated mitochondria provide cellular memory of hematopoietic stem cell replicative history and drive HSC attrition. Cell Stem Cell.

[bib46] Höfer T, Rodewald H-R (2018). Differentiation-based model of hematopoietic stem cell functions and lineage pathways. Blood.

[bib47] Hormaechea-Agulla D, Matatall KA, Le DT, Kain B, Long X, Kus P, Jaksik R, Challen GA, Kimmel M, King KY (2021). Chronic infection drives Dnmt3a-loss-of-function clonal hematopoiesis via IFNγ signaling. Cell Stem Cell.

[bib48] Jaiswal S, Ebert BL (2019). Clonal hematopoiesis in human aging and disease. Science.

[bib49] Jentho E, Ruiz-Moreno C, Novakovic B, Kourtzelis I, Megchelenbrink WL, Martins R, Chavakis T, Soares MP, Kalafati L, Guerra J, Roestel F, Bohm P, Godmann M, Grinenko T, Eugster A, Beretta M, Joosten LAB, Netea MG, Bauer M, Stunnenberg HG, Weis S (2021). Trained innate immunity, long-lasting epigenetic modulation, and skewed myelopoiesis by heme. PNAS.

[bib50] Johansson A, Lin DS, Mercier FE, Yamashita M, Divangahi M, Sieweke MH (2023). Trained immunity and epigenetic memory in long-term self-renewing hematopoietic cells. Experimental Hematology.

[bib51] Kain BN, Tran BT, Luna PN, Cao R, Le DT, Florez MA, Maneix L, Toups JD, Morales-Mantilla DE, Koh S, Han H, Jaksik R, Huang Y, Catic A, Shaw CA, King KY (2023). Hematopoietic stem and progenitor cells confer cross-protective trained immunity in mouse models. iScience.

[bib52] Kalafati L, Kourtzelis I, Schulte-Schrepping J, Li X, Hatzioannou A, Grinenko T, Hagag E, Sinha A, Has C, Dietz S, de Jesus Domingues AM, Nati M, Sormendi S, Neuwirth A, Chatzigeorgiou A, Ziogas A, Lesche M, Dahl A, Henry I, Subramanian P, Wielockx B, Murray P, Mirtschink P, Chung KJ, Schultze JL, Netea MG, Hajishengallis G, Verginis P, Mitroulis I, Chavakis T (2020). Innate immune training of granulopoiesis promotes anti-tumor activity. Cell.

[bib53] Kamada R, Yang W, Zhang Y, Patel MC, Yang Y, Ouda R, Dey A, Wakabayashi Y, Sakaguchi K, Fujita T, Tamura T, Zhu J, Ozato K (2018). Interferon stimulation creates chromatin marks and establishes transcriptional memory. PNAS.

[bib54] Kang YA, Paik H, Zhang SY, Chen JJ, Olson OC, Mitchell CA, Collins A, Swann JW, Warr MR, Fan R, Passegué E (2023). Secretory MPP3 reinforce myeloid differentiation trajectory and amplify myeloid cell production. The Journal of Experimental Medicine.

[bib55] Kang M, Park HK, Kim KS, Choi D (2024). Animal models for transplant immunology: bridging bench to bedside. Clinical Transplantation and Research.

[bib56] Kaufmann E, Sanz J, Dunn JL, Khan N, Mendonça LE, Pacis A, Tzelepis F, Pernet E, Dumaine A, Grenier JC, Mailhot-Léonard F, Ahmed E, Belle J, Besla R, Mazer B, King IL, Nijnik A, Robbins CS, Barreiro LB, Divangahi M (2018). BCG educates hematopoietic stem cells to generate protective innate immunity against Tuberculosis. Cell.

[bib57] Kaufmann E, Khan N, Tran KA, Ulndreaj A, Pernet E, Fontes G, Lupien A, Desmeules P, McIntosh F, Abow A, Moorlag SJCFM, Debisarun P, Mossman K, Banerjee A, Karo-Atar D, Sadeghi M, Mubareka S, Vinh DC, King IL, Robbins CS, Behr MA, Netea MG, Joubert P, Divangahi M (2022). BCG vaccination provides protection against IAV but not SARS-CoV-2. Cell Reports.

[bib58] Khan N, Downey J, Sanz J, Kaufmann E, Blankenhaus B, Pacis A, Pernet E, Ahmed E, Cardoso S, Nijnik A, Mazer B, Sassetti C, Behr MA, Soares MP, Barreiro LB, Divangahi M (2020). *M. tuberculosis* reprograms hematopoietic stem cells to limit myelopoiesis and impair trained immunity. Cell.

[bib59] Khan N, Tran KA, Chevre R, Locher V, Richter M, Sun S, Sadeghi M, Pernet E, Herrero-Cervera A, Grant A, Saif A, Downey J, Kaufmann E, Khader SA, Joubert P, Barreiro LB, Yipp BG, Soehnlein O, Divangahi M (2025). β-Glucan reprograms neutrophils to promote disease tolerance against influenza A virus. Nature Immunology.

[bib60] King KY, Goodell MA (2011). Inflammatory modulation of HSCs: viewing the HSC as a foundation for the immune response. Nature Reviews. Immunology.

[bib61] Kleinnijenhuis J, Quintin J, Preijers F, Benn CS, Joosten LAB, Jacobs C, van Loenhout J, Xavier RJ, Aaby P, van der Meer JWM, van Crevel R, Netea MG (2014). Long-lasting effects of BCG vaccination on both heterologous Th1/Th17 responses and innate trained immunity. Journal of Innate Immunity.

[bib62] Larsen SB, Cowley CJ, Fuchs E (2020a). Epithelial cells: liaisons of immunity. Current Opinion in Immunology.

[bib63] Larsen ES, Joensen UN, Poulsen AM, Goletti D, Johansen IS (2020b). Bacillus Calmette-Guérin immunotherapy for bladder cancer: a review of immunological aspects, clinical effects and BCG infections. APMIS.

[bib64] Le DT, Florez MA, Kus P, Tran BT, Kain B, Zhu Y, Christensen K, Jain A, Malovannaya A, King KY (2023). BATF2 promotes HSC myeloid differentiation by amplifying IFN response mediators during chronic infection. iScience.

[bib65] Li X, Wang H, Yu X, Saha G, Kalafati L, Ioannidis C, Mitroulis I, Netea MG, Chavakis T, Hajishengallis G (2022). Maladaptive innate immune training of myelopoiesis links inflammatory comorbidities. Cell.

[bib66] Li L, Bowling S, McGeary SE, Yu Q, Lemke B, Alcedo K, Jia Y, Liu X, Ferreira M, Klein AM, Wang SW, Camargo FD (2023). A mouse model with high clonal barcode diversity for joint lineage, transcriptomic, and epigenomic profiling in single cells. Cell.

[bib67] Liebermann DA, Gregory B, Hoffman B (1998). AP-1 (Fos/Jun) transcription factors in hematopoietic differentiation and apoptosis. International Journal of Oncology.

[bib68] Matatall KA, Shen C-C, Challen GA, King KY (2014). Type II interferon promotes differentiation of myeloid-biased hematopoietic stem cells. Stem Cells.

[bib69] Matatall KA, Jeong M, Chen S, Sun D, Chen F, Mo Q, Kimmel M, King KY (2016). Chronic infection depletes hematopoietic stem cells through stress-induced terminal differentiation. Cell Reports.

[bib70] Mende N, Laurenti E (2021). Hematopoietic stem and progenitor cells outside the bone marrow: where, when, and why. Experimental Hematology.

[bib71] Mills TS, Kain B, Burchill MA, Danis E, Lucas ED, Culp-Hill R, Cowan CM, Schleicher WE, Patel SB, Tran BT, Cao R, Goodspeed A, Ferrara S, Bevers S, Jirón Tamburini BA, Roede JR, D’Alessandro A, King KY, Pietras EM (2024). A distinct metabolic and epigenetic state drives trained immunity in HSC-derived macrophages from autoimmune mice. Cell Stem Cell.

[bib72] Missinato MA, Murphy S, Lynott M, Yu MS, Kervadec A, Chang Y-L, Kannan S, Loreti M, Lee C, Amatya P, Tanaka H, Huang C-T, Puri PL, Kwon C, Adams PD, Qian L, Sacco A, Andersen P, Colas AR (2023). Conserved transcription factors promote cell fate stability and restrict reprogramming potential in differentiated cells. Nature Communications.

[bib73] Mitroulis I, Ruppova K, Wang B, Chen LS, Grzybek M, Grinenko T, Eugster A, Troullinaki M, Palladini A, Kourtzelis I, Chatzigeorgiou A, Schlitzer A, Beyer M, Joosten LAB, Isermann B, Lesche M, Petzold A, Simons K, Henry I, Dahl A, Schultze JL, Wielockx B, Zamboni N, Mirtschink P, Coskun Ü, Hajishengallis G, Netea MG, Chavakis T (2018). Modulation of myelopoiesis progenitors is an integral component of trained immunity. Cell.

[bib74] Mitroulis I, Kalafati L, Bornhäuser M, Hajishengallis G, Chavakis T (2020). Regulation of the bone marrow niche by inflammation. Frontiers in Immunology.

[bib75] Moorlag SJCFM, Khan N, Novakovic B, Kaufmann E, Jansen T, van Crevel R, Divangahi M, Netea MG (2020). β-Glucan induces protective trained immunity against *Mycobacterium tuberculosis* infection: a key role for IL-1. Cell Reports.

[bib76] Morrison SJ, Scadden DT (2014). The bone marrow niche for haematopoietic stem cells. Nature.

[bib77] Nagai Y, Garrett KP, Ohta S, Bahrun U, Kouro T, Akira S, Takatsu K, Kincade PW (2006). Toll-like receptors on hematopoietic progenitor cells stimulate innate immune system replenishment. Immunity.

[bib78] Naik S, Fuchs E (2022). Inflammatory memory and tissue adaptation in sickness and in health. Nature.

[bib79] Nakauchi H, Takano H, Ema H, Osawa M (1999). Further characterization of CD34-low/negative mouse hematopoietic stem cells. Annals of the New York Academy of Sciences.

[bib80] Netea MG, Domínguez-Andrés J, Barreiro LB, Chavakis T, Divangahi M, Fuchs E, Joosten LAB, van der Meer JWM, Mhlanga MM, Mulder WJM, Riksen NP, Schlitzer A, Schultze JL, Stabell Benn C, Sun JC, Xavier RJ, Latz E (2020). Defining trained immunity and its role in health and disease. Nature Reviews. Immunology.

[bib81] Notta F, Zandi S, Takayama N, Dobson S, Gan OI, Wilson G, Kaufmann KB, McLeod J, Laurenti E, Dunant CF, McPherson JD, Stein LD, Dror Y, Dick JE (2016). Distinct routes of lineage development reshape the human blood hierarchy across ontogeny. Science.

[bib82] Ochando J, Mulder WJM, Madsen JC, Netea MG, Duivenvoorden R (2023). Trained immunity - basic concepts and contributions to immunopathology. Nature Reviews. Nephrology.

[bib83] Ogonek J, Kralj Juric M, Ghimire S, Varanasi PR, Holler E, Greinix H, Weissinger E (2016). Immune reconstitution after allogeneic hematopoietic stem cell transplantation. Frontiers in Immunology.

[bib84] Oguro H, Ding L, Morrison SJ (2013). SLAM family markers resolve functionally distinct subpopulations of hematopoietic stem cells and multipotent progenitors. Cell Stem Cell.

[bib85] Orkin SH, Zon LI (2008). Hematopoiesis: an evolving paradigm for stem cell biology. Cell.

[bib86] Patel AA, Zhang Y, Fullerton JN, Boelen L, Rongvaux A, Maini AA, Bigley V, Flavell RA, Gilroy DW, Asquith B, Macallan D, Yona S (2017). The fate and lifespan of human monocyte subsets in steady state and systemic inflammation. The Journal of Experimental Medicine.

[bib87] Pei W, Feyerabend TB, Rössler J, Wang X, Postrach D, Busch K, Rode I, Klapproth K, Dietlein N, Quedenau C, Chen W, Sauer S, Wolf S, Höfer T, Rodewald HR (2017). Polylox barcoding reveals haematopoietic stem cell fates realized in vivo. Nature.

[bib88] Pietras EM, Reynaud D, Kang YA, Carlin D, Calero-Nieto FJ, Leavitt AD, Stuart JM, Göttgens B, Passegué E (2015). Functionally distinct subsets of lineage-biased multipotent progenitors control blood production in normal and regenerative conditions. Cell Stem Cell.

[bib89] Pietras EM (2017). Inflammation: a key regulator of hematopoietic stem cell fate in health and disease. Blood.

[bib90] Quintin J, Saeed S, Martens JHA, Giamarellos-Bourboulis EJ, Ifrim DC, Logie C, Jacobs L, Jansen T, Kullberg BJ, Wijmenga C, Joosten LAB, Xavier RJ, van der Meer JWM, Stunnenberg HG, Netea MG (2012). Candida albicans infection affords protection against reinfection via functional reprogramming of monocytes. Cell Host & Microbe.

[bib91] Rathinam C, Poueymirou WT, Rojas J, Murphy AJ, Valenzuela DM, Yancopoulos GD, Rongvaux A, Eynon EE, Manz MG, Flavell RA (2011). Efficient differentiation and function of human macrophages in humanized CSF-1 mice. Blood.

[bib92] Riksen NP, Netea MG (2021). Immunometabolic control of trained immunity. Molecular Aspects of Medicine.

[bib93] Rix B, Maduro AH, Bridge KS, Grey W (2022). Markers for human haematopoietic stem cells: The disconnect between an identification marker and its function. Frontiers in Physiology.

[bib94] Saeed S, Quintin J, Kerstens HHD, Rao NA, Aghajanirefah A, Matarese F, Cheng S-C, Ratter J, Berentsen K, van der Ent MA, Sharifi N, Janssen-Megens EM, Ter Huurne M, Mandoli A, van Schaik T, Ng A, Burden F, Downes K, Frontini M, Kumar V, Giamarellos-Bourboulis EJ, Ouwehand WH, van der Meer JWM, Joosten LAB, Wijmenga C, Martens JHA, Xavier RJ, Logie C, Netea MG, Stunnenberg HG (2014). Epigenetic programming of monocyte-to-macrophage differentiation and trained innate immunity. Science.

[bib95] Sawai CM, Babovic S, Upadhaya S, Knapp DJHF, Lavin Y, Lau CM, Goloborodko A, Feng J, Fujisaki J, Ding L, Mirny LA, Merad M, Eaves CJ, Reizis B (2016). Hematopoietic stem cells are the major source of multilineage hematopoiesis in adult animals. Immunity.

[bib96] Säwen P, Eldeeb M, Erlandsson E, Kristiansen TA, Laterza C, Kokaia Z, Karlsson G, Yuan J, Soneji S, Mandal PK, Rossi DJ, Bryder D (2018). Murine HSCs contribute actively to native hematopoiesis but with reduced differentiation capacity upon aging. eLife.

[bib97] Shaban D, Najm N, Droin L, Nijnik A (2025). Hematopoietic stem cell fates and the cellular hierarchy of mammalian hematopoiesis: from transplantation models to new insights from in situ analyses. Stem Cell Reviews and Reports.

[bib98] Spangrude GJ, Heimfeld S, Weissman IL (1988). Purification and characterization of mouse hematopoietic stem cells. Science.

[bib99] Sun J, Ramos A, Chapman B, Johnnidis JB, Le L, Ho YJ, Klein A, Hofmann O, Camargo FD (2014). Clonal dynamics of native haematopoiesis. Nature.

[bib100] Sun SJ, Aguirre-Gamboa R, de Bree LCJ, Sanz J, Dumaine A, van der Velden W, Joosten LAB, Khader S, Divangahi M, Netea MG, Barreiro LB (2024). BCG vaccination alters the epigenetic landscape of progenitor cells in human bone marrow to influence innate immune responses. Immunity.

[bib101] Takizawa H, Boettcher S, Manz MG (2012). Demand-adapted regulation of early hematopoiesis in infection and inflammation. Blood.

[bib102] Taylor SJ, Stauber J, Bohorquez O, Tatsumi G, Kumari R, Chakraborty J, Bartholdy BA, Schwenger E, Sundaravel S, Farahat AA, Wheat JC, Goldfinger M, Verma A, Kumar A, Boykin DW, Stengel KR, Poon GMK, Steidl U (2024). Pharmacological restriction of genomic binding sites redirects PU.1 pioneer transcription factor activity. Nature Genetics.

[bib103] Teh YC, Ding JL, Ng LG, Chong SZ (2019). Capturing the fantastic voyage of monocytes through time and space. Frontiers in Immunology.

[bib104] van Puffelen JH, Keating ST, Oosterwijk E, van der Heijden AG, Netea MG, Joosten LAB, Vermeulen SH (2020). Trained immunity as a molecular mechanism for BCG immunotherapy in bladder cancer. Nature Reviews. Urology.

[bib105] Verma D, Parasa VR, Raffetseder J, Martis M, Mehta RB, Netea M, Lerm M (2017). Anti-mycobacterial activity correlates with altered DNA methylation pattern in immune cells from BCG-vaccinated subjects. Scientific Reports.

[bib106] Walk J, de Bree LCJ, Graumans W, Stoter R, van Gemert G-J, van de Vegte-Bolmer M, Teelen K, Hermsen CC, Arts RJW, Behet MC, Keramati F, Moorlag SJCFM, Yang ASP, van Crevel R, Aaby P, de Mast Q, van der Ven AJAM, Stabell Benn C, Netea MG, Sauerwein RW (2019). Outcomes of controlled human malaria infection after BCG vaccination. Nature Communications.

[bib107] Walk J, Keramati F, de Bree LCJ, Arts RJW, Blok B, Netea MG, Stunnenberg HG, Sauerwein RW (2020). Controlled human malaria infection induces long-term functional changes in monocytes. Frontiers in Molecular Biosciences.

[bib108] Wang T, Nandakumar V, Jiang XX, Jones L, Yang AG, Huang XF, Chen SY (2013). The control of hematopoietic stem cell maintenance, self-renewal, and differentiation by Mysm1-mediated epigenetic regulation. Blood.

[bib109] Wilkinson AC, Ishida R, Kikuchi M, Sudo K, Morita M, Crisostomo RV, Yamamoto R, Loh KM, Nakamura Y, Watanabe M, Nakauchi H, Yamazaki S (2019). Long-term ex vivo haematopoietic-stem-cell expansion allows nonconditioned transplantation. Nature.

[bib110] Wilson A, Laurenti E, Oser G, van der Wath RC, Blanco-Bose W, Jaworski M, Offner S, Dunant CF, Eshkind L, Bockamp E, Lió P, Macdonald HR, Trumpp A (2008). Hematopoietic stem cells reversibly switch from dormancy to self-renewal during homeostasis and repair. Cell.

[bib111] Wu YL, Lin ZJ, Li CC, Lin X, Shan SK, Guo B, Zheng MH, Li F, Yuan LQ, Li Z (2023). Epigenetic regulation in metabolic diseases: mechanisms and advances in clinical study. Signal Transduction and Targeted Therapy.

[bib112] Yang L, Bryder D, Adolfsson J, Nygren J, Månsson R, Sigvardsson M, Jacobsen SEW (2005). Identification of Lin–Sca1+kit+CD34+Flt3– short-term hematopoietic stem cells capable of rapidly reconstituting and rescuing myeloablated transplant recipients. Blood.

[bib113] Yang Y, Gomez N, Infarinato N, Adam RC, Sribour M, Baek I, Laurin M, Fuchs E (2023). The pioneer factor SOX9 competes for epigenetic factors to switch stem cell fates. Nature Cell Biology.

[bib114] Yao Y, Jeyanathan M, Haddadi S, Barra NG, Vaseghi-Shanjani M, Damjanovic D, Lai R, Afkhami S, Chen Y, Dvorkin-Gheva A, Robbins CS, Schertzer JD, Xing Z (2018). Induction of autonomous memory alveolar macrophages requires T cell help and is critical to trained immunity. Cell.

[bib115] Yeung J, Liao A, Shaw M, Silva S, Vetharoy W, Rico DL, Kirby I, Zammarchi F, Havenith K, de Haan L, van Berkel PH, Sebire N, Ogunbiyi OK, Booth C, Gaspar HB, Thrasher AJ, Chester KA, Amrolia PJ (2024). Anti-CD45 PBD-based antibody-drug conjugates are effective targeted conditioning agents for gene therapy and stem cell transplant. Molecular Therapy.

[bib116] Zeng AGX, Nagree MS, Jakobsen NA, Shah S, Murison A (2023). A hematopoietic stem cell subset that retains memory of prior inflammatory stress accumulates in aging and clonal hematopoiesis. bioRxiv.

[bib117] Zhang C, Chen Y, Sun B, Wang L, Yang Y, Ma D, Lv J, Heng J, Ding Y, Xue Y, Lu X, Xiao W, Yang YG, Liu F (2017). m6A modulates haematopoietic stem and progenitor cell specification. Nature.

[bib118] Zhu Y, Gao Q, Zhang J, Cheng Y, Yang S, Xu R, Yuan J, Novakovic B, Netea MG, Cheng SC (2024). Persistent bone marrow hemozoin accumulation confers a survival advantage against bacterial infection via cell-intrinsic Myd88 signaling. Cell Reports.

[bib119] Ziogas A, Novakovic B, Ventriglia L, Galang N, Tran KA, Li W, Matzaraki V, van Unen N, Schlüter T, Ferreira AV, Moorlag S, Koeken V, Moyo M, Li X, Baltissen MPA, Martens JHA, Li Y, Divangahi M, Joosten LAB, Mhlanga MM, Netea MG (2025). Long-term histone lactylation connects metabolic and epigenetic rewiring in innate immune memory. Cell.

